# Prostate Cancer Risk Inflation as a Consequence of Image-targeted Biopsy of the Prostate: A Computer Simulation Study

**DOI:** 10.1016/j.eururo.2012.12.057

**Published:** 2014-03

**Authors:** Nicola L. Robertson, Yipeng Hu, Hashim U. Ahmed, Alex Freeman, Dean Barratt, Mark Emberton

**Affiliations:** a Department of Radiology, Royal Free Hospital, London, UK; b UCL Centre for Medical Image Computing, Department of Medical Physics and Bioengineering, University College London, London, UK; c Department of Urology, University College London Hospital Trust, London, UK; d Division of Surgery and Interventional Science, University College London, London, UK; e Department of Histopathology, University College London Hospital Trust, London, UK

**Keywords:** Prostate, Biopsy, Simulation, Risk

## Abstract

**Background:**

Prostate biopsy parameters are commonly used to attribute cancer risk. A targeted approach to lesions found on imaging may have an impact on the risk attribution given to a man.

**Objective:**

To evaluate whether, based on computer simulation, targeting of lesions during biopsy results in reclassification of cancer risk when compared with transrectal ultrasound (TRUS) guided biopsy.

**Design, setting, and participants:**

A total of 107 reconstructed three-dimensional models of whole-mount radical prostatectomy specimens were used for computer simulations. Systematic 12-core TRUS biopsy was compared with transperineal targeted biopsies using between one and five cores. All biopsy strategies incorporated operator and needle deflection error. A target was defined as any lesion ≥0.2 ml. A false-positive magnetic resonance imaging identification rate of 34% was applied.

**Outcome measurements and statistical analysis:**

Sensitivity was calculated for the detection of all cancer and clinically significant disease. Cases were designated as high risk based on achieving ≥6 mm cancer length and/or ≥50% positive cores. Statistical significance (*p* values) was calculated using both a paired Kolmogorov-Smirnov test and the *t* test.

**Results and limitations:**

When applying a widely used biopsy criteria to designate risk, 12-core TRUS biopsy classified only 24% (20 of 85) of clinically significant cases as high risk, compared with 74% (63 of 85) of cases using 4 targeted cores. The targeted strategy reported a significantly higher proportion of positive cores (44% vs 11%; *p* < 0.0001) and a significantly greater mean maximum cancer core length (7.8 mm vs 4.3 mm; *p* < 0.0001) when compared with 12-core TRUS biopsy. Computer simulations may not reflect the sources of errors encountered in clinical practice. To mitigate this we incorporated all known major sources of error to maximise clinical relevance.

**Conclusions:**

Image-targeted biopsy results in an increase in risk attribution if traditional criteria, based on cancer core length and the proportion of positive cores, are applied. Targeted biopsy strategies will require new risk stratification models that account for the increased likelihood of sampling the tumour.

## Take Home Message

Image-directed prostate biopsy results in an increase in risk attribution if traditional criteria (maximum cancer core length, proportion of positive cores) are applied. Targeted biopsy strategies will require new risk stratification models accounting for the increased likelihood of sampling the tumour.

## 1 Introduction

The current diagnostic pathway in prostate cancer relies on the transrectal ultrasound (TRUS) guided prostate biopsy test, applied after a man presents with an elevated serum prostate-specific antigen. The random and systematic errors that occur when this test is conducted blind to the location of a cancer have been widely discussed [1], [2], [3].

State-of-the-art imaging such as multiparametric magnetic resonance imaging (mpMRI) [4] or novel ultrasound (US) techniques [5] could overcome these errors by providing information on the location and size of suspicious lesions, thus allowing such lesions to be targeted.

Biopsy data are commonly used to determine cancer risk. A targeted approach to lesions found on imaging may have an impact on the risk attributed to a particular man. Features widely used to indicate high risk include Gleason score ≥7, as well as parameters to indicate the extent of cancer such as maximum cancer core length (MCCL), maximum percentage cancer, and the number of positive biopsies [6]. However, if a tumour is exposed to a greater sampling density than the rest of the prostate, it is likely that the proportion of cores that are positive and the MCCL will be greater compared with a TRUS biopsy. In addition, higher Gleason patterns, if truly present, are more likely to be sampled.

The aims of this study were to establish whether, and the extent to which, the phenomenon of risk escalation occurs in men who undergo targeted biopsy, by means of a computer simulation.

## 2 Materials and methods

From 1999 to 2001, 107 consecutive radical prostatectomy whole-mount specimens that underwent 5-mm step sectioning according to the Stanford protocol were analysed [7]. A single histopathologist contoured all cancer foci by hand on each pathology slide. For each slice, the prostate capsule and tumour contours were scanned and digitised using a flatbed scanner. A three-dimensional (3D) computer model/image reconstruction was produced for each gland using custom-written computer software. The scanned two-dimensional cross sections were first aligned. Image registration and a shape-based interpolation method matched the adjacent gland slices to the chosen midgland reference slice [8], [9], [10], [11]. A data-specific correction factor was applied to estimate, and thereafter reverse, the fixation-related tissue shrinkage effect [11]. This correction factor, calculated from measurements obtained before and after formalin fixation, was 1.10 (equivalent to a 33% increase in volume), assumed to be isotropic, and applied to all specimens. The detailed methodology for this 3D reconstruction was previously described [12], [13].

A false-positive rate for prostate mpMRI was incorporated. This was based on a study recently published [14], in which image-targeted biopsies were performed in 182 men with a lesion suspicious for prostate cancer on mpMRI. MRI false positives are the result of an MRI signal that is incorrect, a targeting miss, or a tissue capture failure. The study demonstrated a 34% mpMRI false-positive rate. Applying this rate to our simulation resulted in a total of 141 prostates for biopsy.

A false-negative rate for prostate mpMRI was not incorporated because men with no lesion on mpMRI have no target for biopsy and therefore revert to the standard of care, the TRUS biopsy.

It was previously demonstrated that lesions ≥0.2 ml in volume on mpMRI can be detected with 77% sensitivity and 91% specificity [15]; therefore, we defined a target as any lesion ≥0.2 ml.

### 2.1 Simulated biopsy

For each prostate model, 500 simulations of each biopsy strategy were performed. The biopsy strategies included a 12-core TRUS biopsy and transperineal targeted biopsies. In practice the number of image-targeted cores taken depends on the clinical context and the operator performing the biopsy. Therefore, each simulated transperineal targeted scheme was repeated five times per prostate model, with the number of targeted cores deployed ranging from one in the first series to five in the fifth.

**Fig. 1:**
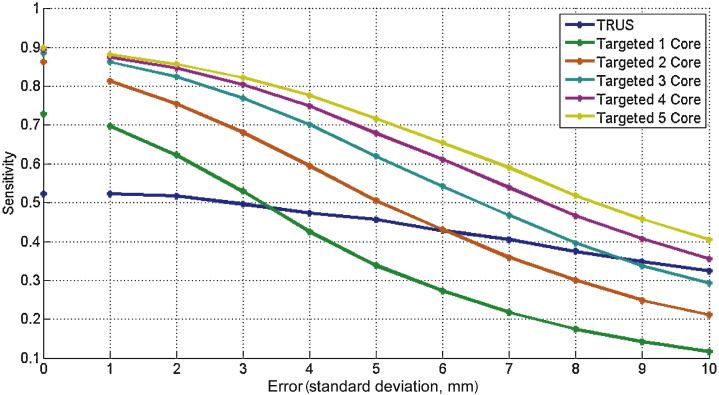
All-cancer sensitivity of biopsy simulations with increasing error.

**Fig. 2:**
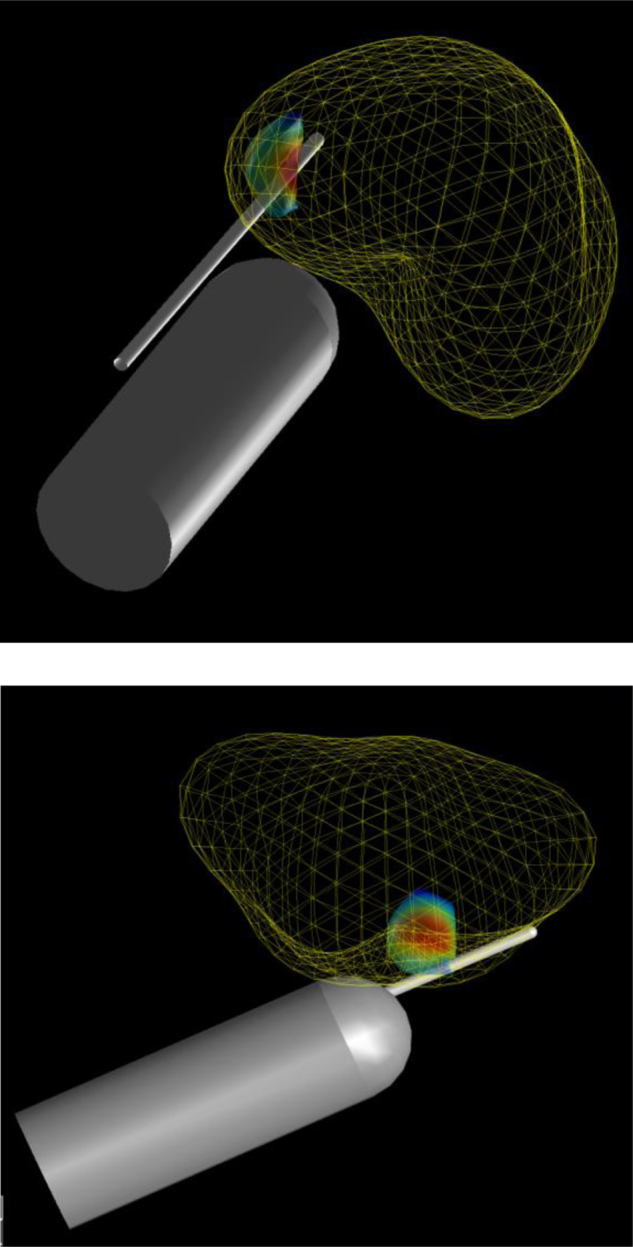
Simulated 12-core transrectal ultrasound biopsy. Correct anatomic position and orientation estimated using T2-weighted magnetic resonance imaging (1.5 T). Three-dimensional coordinates taken from the centre of the anus and gland, and aligned with the base-apex axis. Coordinates were used to approximate the variability in the probe/needle insertion location and trajectory during simulated biopsies. Simulated needle insertions were automatically calculated by computer software, so the conduct of the biopsies was fully blinded to the pathology.

Errors were incorporated for all simulations to reflect registration (or operator) deficiencies and needle deflection. In clinical practice, the total targeting error equates to the sum of these two errors [16]. All biopsy strategies were performed with a range of applied error, from 1 mm to 10 mm (Fig. 1); however, to ensure the results generated were comparable, we set our total targeting error at 5 mm. This error was calculated using (1) a needle deflection error with standard deviation (SD) of 3 mm in any direction (measured at the midpoint of the effective part of the needle), in accordance with previous studies [12], [13], and (2) a registration error with an SD of ±3 mm (Fig. 2), which represents the misalignment between MRI and TRUS images (ie, US-MRI registration error) [17], [18], [19]. The set total variance of the error (25 mm^2^) was larger than the additive variance (9 + 9 mm^2^). All errors were assumed to have zero-mean normally distributed components in the three orthogonal directions.

### 2.2 Twelve-core transrectal ultrasound biopsy

For us to approximate the variability in the probe and needle trajectory during TRUS biopsies, we estimated the anatomic position and orientation of the prostate gland relative to the rectum using T2-weighted MRI (1.5 T) sequences of patients with prostate cancer. Three-dimensional coordinates were taken from the centre of the anus and gland, and aligned with the base-apex axis. This information allowed simulated needle insertions to be performed automatically by the computer software, so the conduct of the biopsies was fully blinded to the pathology, as would occur in clinical practice (Fig. 2).

### 2.3 Transperineal targeted biopsy

**Fig. 3:**
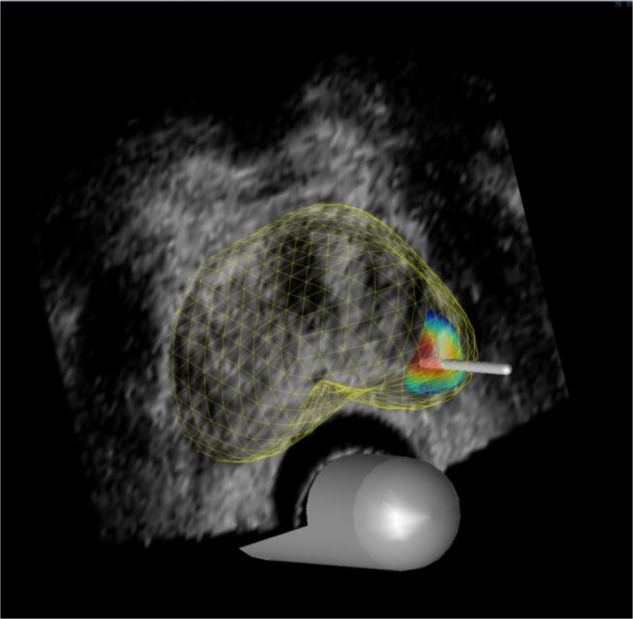
Simulated targeted biopsy. Using a 5-mm brachytherapy template, visible urethra was aligned to the D 2.0 grid coordinate on midgland transverse view. Planned positions of the targeted cores (1–5) were optimised so that the total cancer core length obtained was maximised.

Using a 5-mm brachytherapy template, the visible urethra was aligned to the D 2.0 grid coordinate on a midgland transverse view (Fig. 3). The planned positions of the targeted cores were optimised based on a method developed in our research group; by aiming for the deepest part of the lesion, the total cancer core length obtained was maximised.

### 2.4 Analyses

The MCCL and percentage of positive cores were evaluated for each biopsy strategy. In our institution, three to four image-directed targeted cores are typically taken per lesion. We have therefore focussed our results to compare 12-core TRUS biopsy with the 3-core and 4-core targeted biopsy strategies. Cases were designated as low or high risk according to criteria detailed in Table 1.

**Table 1:** Simulation biopsy risk stratification criteria

Risk	Histopathology criteria
High	• ≥6 mm maximum cancer core length
	• ≥50% positive cores
Low	• <6 mm maximum cancer core length
	• <50% positive cores

### 2.5 Statistics

Sensitivity was calculated for each biopsy strategy for the detection of all cancer and clinically significant disease (lesion size ≥0.5 ml and/or Gleason grade ≥7). The impact of increasing the error was evaluated (Fig. 1), as well as the proportion of detected cases that were attributed a high-risk status (based on biopsy parameters) by each biopsy strategy (Table 1). Statistical significance (*p* value) was calculated using the Student *t* test and a paired Kolmogorov-Smirnov test [20] (significance level α = 0.05) because the MCCL and percentage of positive cores have a skewed distribution.

## 3 Results

**Fig. 4:**
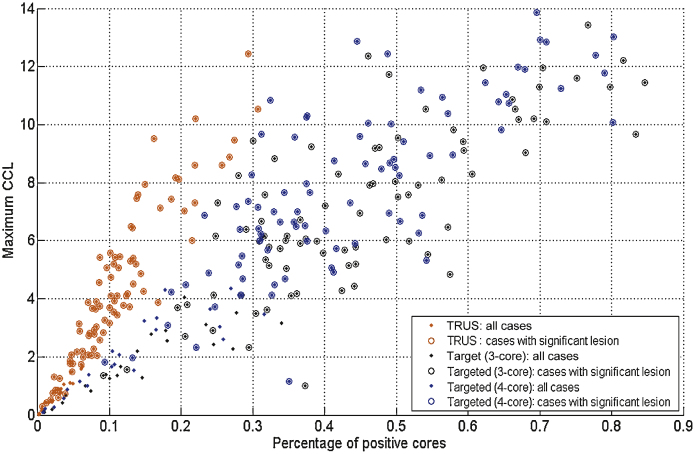
Detection of all cancer, and clinically significant disease, using 12-core transrectal ultrasound (TRUS) biopsy and a 3- or 4-core targeted strategy. CCL = cancer core length.

Table 2 shows the baseline characteristics of prostate cancer cases used in the simulation. Setting the total targeted error at 5 mm, the sensitivity for all cancer detection was 0.46, 0.62, and 0.68 for the 12-core TRUS, 3-core, and 4-core targeted biopsy strategies, respectively (Fig. 1). The 12-core TRUS biopsy detected 91% (77 of 85) of clinically significant cancers compared with 98% (83 of 85) and 99% (84 of 85) detected using three or four targeted cores, respectively (Fig. 4).

**Table 2:** Baseline characteristics for prostate cases used in the simulation

Characteristic, median (mean, SD, range)	Value
Age, yr	62 (61.1, 6.4, 44–74)
PSA concentration, ng/ml	8.5 (9.7, 5.9, 0.8–36.2)
Gleason score, % (*n*)	
≤6	57 (61)
7	35 (37)
≥8	8 (9)
Pathologic stage, % (*n*)	
pT2a	7.5 (8)
pT2b	2 (2)
pT2c	49.5 (53)
pT3a	33.6 (36)
pT3b	5.6 (6)
pT4	2 (2)
Risk groups, NCCN classification, % (*n*)	
Low	5.6 (6)
Intermediate	47.7 (51)
High	46.7 (50)
Prostate volume, ml, median (range)	50.2 (26.8–127.7)
No. of lesions	
Anterior	415
Posterior	250
Full cohort	
≥0.2 ml	149
≥0.5 ml	97
Low to intermediate risk	
≥0.2 ml	68
≥0.5 ml	43
Lesions per prostate, median (range)	5 (1–21)
Lesion volumes, ml, median (mean, SD, range)	
All (*n* = 665)	0.031 (0.374, 1.110, 0.001–13.242)
Index (*n* = 107)	1.215 (1.895, 2.176, 0.015–13.242)
Nonindex (*n* = 558)	0.019 (0.082, 0.343, 0.001–1.842)

NCCN = National Comprehensive Cancer Network; PSA = prostate-specific antigen; SD = standard deviation.

The targeted biopsies reported a significantly higher proportion of positive cores (*p* < 0.0001) and significantly greater MCCLs (*p* < 0.0001) (Table 3). When all cases with cancer were considered, the mean MCCL was 2.7 mm, 4.6 mm, and 5.1 mm, and the proportion of positive cores was 7%, 29%, and 28% for the 12-core TRUS biopsy, 3-core targeted, and 4-core targeted biopsies, respectively. When only considering cases that were clinically significant on whole-mount histology, mean MCCL was 4.3 mm, 7.2 mm, and 7.8 mm (*p* < 0.0001), and the proportion of positive cores was 11%, 45%, and 44% for the 12-core TRUS biopsy, 3-core targeted, and 4-core targeted schemes, respectively (*p* < 0.0001).

**Table 3:** Proportion of positive cores and maximum cancer core lengths for transrectal ultrasound biopsy and three- or four-core targeted biopsy*

	Maximum cancer core length, mm			Positive cores, %		
	Mean ± SD	Median	90th percentile	Mean ± SD	Median	90th percentile
All data						
TRUS 12 core	2.7 ± 2.9	1.9	7.4	7.1 ± 7.2	5.7	16.9
Targeted 3 core	4.6 ± 3.9	4.3	10.2	29.3 ± 24.1	30.9	63.7
Targeted 4 core	5.1 ± 4.2	4.9	11.0	28.2 ± 23.3	29.3	63.1
High risk (index volume ≥0.5 or Gleason≥7)						
TRUS 12 core	4.3 ± 2.7	3.9	8.2	11.0 ± 6.7	10.1	21.5
Targeted 3 core	7.2 ± 2.8	6.7	11.3	45.0 ± 16.9	42.7	69.9
Targeted 4 core	7.8 ± 2.9	7.4	11.9	43.5 ± 16.3	41.0	68.0
Low risk (index volume <0.5 and Gleason <7)						
TRUS 12 core	0.3 ± 0.5	0.0	1.0	1.0 ± 1.7	0.0	3.6
Targeted 3 core	0.7 ± 1.1	0.0	2.7	5.4 ± 9.0	0.0	20.2
Targeted 4 core	0.8 ± 1.3	0.0	3.2	5.1 ± 8.5	0.0	17.6

SD = standard deviation; TRUS = transrectal ultrasound.

* For all cancer, clinically significant disease, and clinically insignificant disease.

When applying biopsy risk stratification criteria (≥6 mm MCCL and/or ≥50% positive cores) (Table 1), the 12-core TRUS biopsy correctly attributed a high-risk classification to 24% (20 of 85) clinically significant cases. This compared with 66% (56 of 85) using the three-core targeted technique and 74% (63 of 85) using the four-core targeted scheme (Fig. 4).

We also evaluated how disease burden would be represented by targeted and TRUS biopsy strategies in those cases that were defined as clinically insignificant (Gleason < 7 and/or lesion size <0.5 ml). All insignificant lesions were attributed a low-risk classification when applying our biopsy risk stratification criteria. However, the targeted biopsy strategies demonstrated a higher disease burden, with mean MCCL of 0.3 mm, 0.7 mm, and 0.8 mm, and the proportion of positive cores 1%, 5%, and 5% for the 12-core TRUS biopsy, 3-core, and 4-core targeted strategy, respectively (Table 3).

## 4 Discussion

Our simulation has shown that image-directed biopsy introduces a systematic increase in risk attribution if risk models derived from conventional TRUS biopsy are applied. This agrees with clinical intuition because currently applied risk stratification schemes are not optimised for men who have a lesion defined on imaging who undergo tumour-targeted biopsies.

### 4.1 Limitations

Before we consider the clinical implications of our results, it is important to recognise certain limitations associated with our method. First, the analysis of Gleason grade is limited in the current study. We defined clinically significant disease as Gleason ≥7 and/or volume ≥0.5 ml but were unable to study the dominance of Gleason pattern 4 in more detail because areas of pattern within a lesion were not accurately mapped.

Second, the use of computer simulations may not reflect true clinical practice. To mitigate for this we incorporated errors to reflect operator/registration deficiencies and needle deflection. In practice, there are three ways in which targeted biopsies can be carried out: US-MRI fusion or registration using overlay software, cognitive registration using TRUS guidance in which the operator effectively eyeballs where the lesion may be as in our recent report, and in-bore MRI targeting. US-MRI fusion registration, and the associated error, has been widely researched [17], [18], [19]. Because fusion software is being used increasingly in clinical practice, we specifically used this error to ensure our results were reproducible and applicable. We acknowledge that cognitive targeting with TRUS would involve applying a much greater—but currently unquantifiable—error. The accuracy of in-bore MRI-guided targeting is also uncertain, but we would expect the error to be comparable or perhaps even lower than using TRUS-MRI fusion, especially if a robot-driven needle placement is used.

Third, TRUS biopsy and a transperineal-targeted strategy differ with respect to needle orientation. This could affect MCCL depending on lesion position and shape. Having said this, there are both ethical and technical limitations to exploring this phenomenon in vivo because there are limits to the number of needle deployments that men will tolerate, and it would be impossible to subject men to repeated testing. Blinding is also difficult due to needle tracking, as is the elimination of the bias associated with order effects.

It is possible to biopsy radical prostatectomy specimens prior to formalin fixation and whole-mount slicing, which may help correct the deficiency in Gleason grade analysis. However, such experiments are subject to various errors that mean differences with the in vivo clinical procedure will still exist. For example, coregistration of biopsy against the processed specimen has numerous methodological problems that include orientation error, gland shrinkage and distortion, tissue loss through trimming, and difficulty with standardising distance measurements in the direction of the needle axis.

On the contrary, simulation may allow us the optimal opportunity to estimate the effect on risk stratification that we can expect if we adopt an image-directed biopsy strategy versus one that is not informed by location.

### 4.2 Clinical implications

The recent growth of interest in and accessibility to mpMRI and novel US techniques as a means of localising prostate cancer has led to image-directed targeted biopsy sampling strategies being increasingly adopted [4], [5]. A recent systematic review of image-guided biopsy versus biopsy blind to location suggests the superiority of the former [4]. In one of the largest studies included within the review, Haffner and colleagues found that image-directed biopsies were associated with a longer MCCL compared with systematic biopsies, with values of 5.6 mm and 4.7 mm, respectively. It was also reported that the image-targeted biopsies were associated with a 16% greater detection of Gleason grade 4/5 than systematic biopsies [21].

If the trend towards image-guided biopsy continues unchecked, it is likely that we will witness a systematic increase in risk attribution in the men subjected to biopsy if the standard criteria for attributing risk are applied.

It is therefore likely that new risk prediction models based on targeted biopsies will be required. As a start to correct what could be regarded as an artefactual increase in cancer risk derived from targeted biopsy, a risk stratification system that is independent of the number of positive cores could be considered. Some of these systems have been validated and confer risk based on Gleason grade and MCCL [13]. In a targeted biopsy that is positive in >50% of the cores obtained from the target region, it is likely that the MCCL is representative of the maximum dimension of the tumour and may be used to infer tumour volume. Recent studies have shown that our threshold for clinical significance should be higher than what was previously acceptable, with calls to raise the volume threshold to 1.3 ml [22]. Incorporating the amount of Gleason pattern 4 into risk models may also derive considerable benefit [23]; such a parameter is likely to be better represented by a targeted biopsy than a TRUS biopsy [24].

One of the benefits of an image-directed strategy is that it confers an upper ceiling of risk if the target is real and the targeting accurate. Information on risk is more useful when presented this way because patients can use this information more readily in their choice of therapy. This compares with TRUS-guided biopsy, which tends to give us information on the lower limit of disease as a result of the random sampling it uses. It is for this reason that men are upgraded on review of radical prostatectomy histopathology or when rebiopsied on active surveillance.

Our computer simulations are intended to explore a concept, namely that of deliberately oversampling a given volume of tissue. We anticipate that more work using ex vivo tissue sampled in a targeted manner will in particular have some utility in exploring the distribution of Gleason patterns when we target versus times when we do not.

## 5 Conclusions

Our computer simulation showed an increase in MCCL and the proportion of cores that were positive when an image-directed biopsy was compared with a non–image-directed biopsy. This could lead to inflation in risk attribution as a consequence of deliberate oversampling of one part of the prostate—in other words targeting. New risk stratification models may be required for men who have pathology derived from image-directed biopsy strategies.

  ***Author contributions:*** Nicola Robertson had full access to all the data in the study and takes responsibility for the integrity of the data and the accuracy of the data analysis.  

*Study concept and design:* Robertson, Hu, Ahmed, Freeman, Barratt, Emberton.

*Acquisition of data:* Robertson, Hu, Ahmed, Freeman.

*Analysis and interpretation of data:* Robertson, Hu, Ahmed, Freeman, Barratt, Emberton.

*Drafting of the manuscript:* Robertson, Hu, Ahmed, Freeman, Barratt, Emberton.

*Critical revision of the manuscript for important intellectual content:* Robertson, Hu, Ahmed, Freeman, Barratt, Emberton.

*Statistical analysis:* Robertson, Hu, Ahmed, Freeman, Barratt, Emberton.

*Obtaining funding:* Robertson, Barratt, Emberton.

*Administrative, technical, or material support:* Robertson, Hu, Ahmed, Freeman, Barratt, Emberton.

*Supervision:* Robertson, Hu, Ahmed, Freeman, Barratt, Emberton.

*Other* (specify): None.  

***Financial disclosures:*** Nicola Robertson certifies that all conflicts of interest, including specific financial interests and relationships and affiliations relevant to the subject matter or materials discussed in the manuscript (eg, employment/affiliation, grants or funding, consultancies, honoraria, stock ownership or options, expert testimony, royalties, or patents filed, received, or pending), are the following: Mark Emberton and Hashim U. Ahmed receive research funding for clinical trials in imaging and therapy of the prostate from Steba Biotech (France), Advanced Medical Diagnostics SAS (Belgium), and USHIFU/Focus Surgery (USA). Mark Emberton is a paid medical consultant to Steba Biotech, USHIFU, and GSK. He receives research support from the UK National Institute of Health Research, UCLH/UCL Comprehensive Biomedical Research Centre, London. Hashim U. Ahmed was previously a paid medical consultant for Steba Biotech (paid into a university discretionary research account) and GE/Oncura plus receives funding from the Medical Research Council (UK). Hashim U. Ahmed and Mark Emberton have both received payments to attend medical conferences from the companies just cited. Dean Barratt is the director of Sageta Ltd. The other authors have nothing to disclose.  

***Funding/Support and role of the sponsor:*** This work was undertaken at UCLH/UCL, which received a proportion of funding from the Department of Health's NIHR Biomedical Research Centres funding scheme (Grant Reference 96). This work was partly supported by The Prostate Cancer Charity (Grant PG10-30). None of the funding sources had any role or input into the design and conduct of the study or approval of the manuscript. They did aid in the collection of data.  

***Acknowledgment statement:*** We would like to thank the urologic and pathologic contributors to the UCLH radical prostatectomy database.
